# Inflammation in Primary and Metastatic Liver Tumorigenesis–Under the Influence of Alcohol and High-Fat Diets

**DOI:** 10.3390/nu12040933

**Published:** 2020-03-27

**Authors:** Lauren S. Strathearn, Afanasii I. Stepanov, Joan Font-Burgada

**Affiliations:** Cancer Biology Program, Fox Chase Cancer Center, Philadelphia, PA 19111, USA; lauren.strathearn@fccc.edu (L.S.S.); afanasii.stepanov@fccc.edu (A.I.S.)

**Keywords:** inflammation, liver, hepatocellular carcinoma, colorectal cancer, liver metastasis, high-fat diet, alcohol, gut–liver axis, gut microbiota

## Abstract

The liver plays an outsized role in oncology. Liver tumors are one of the most frequently found tumors in cancer patients and these arise from either primary or metastatic disease. Hepatocellular carcinoma (HCC), the most prevalent form of primary liver cancer and the 6th most common cancer type overall, is expected to become the 3rd leading cause of cancer mortality in the US by the year 2030. The liver is also the most common site of distant metastasis from solid tumors. For instance, colorectal cancer (CRC) metastasizes to the liver in two-thirds of cases, and CRC liver metastasis is the leading cause of mortality in these patients. The interplay between inflammation and cancer is unmistakably evident in the liver. In nearly every case, HCC is diagnosed in chronic liver disease (CLD) and cirrhosis background. The consumption of a Western-style high-fat diet is a major risk factor for the development of non-alcoholic fatty liver disease (NAFLD) and non-alcoholic steatohepatitis (NASH), both of which are becoming more prevalent in parallel with the obesity epidemic. Excessive alcohol intake also contributes significantly to the CLD burden in the form of alcoholic liver disease (ALD). Inflammation is a key component in the development of all CLDs. Additionally, during the development of liver metastasis, pro-inflammatory signaling is crucial in eliminating invading cancer cells but ironically also helps foster a pro-metastatic environment that supports metastatic seeding and colonization. Here we review how Westernized high-fat diets and excessive alcohol intake can influence inflammation within the liver microenvironment, stimulating both primary and metastatic liver tumorigenesis.

## 1. Introduction

Inflammation is a potent enabler of tumorigenesis; contributing to tumor initiation, tumor growth and facilitating progression to metastasis [1,2]. Acute inflammatory responses are typically triggered by noxious stimuli, such as infection or tissue damage. Chronic inflammation however, is thought to be induced as a result of prolonged tissue malfunction and loss of homeostasis, often in sterile conditions in the absence of invading pathogens. Critically, many conditions associated with persistent underlying inflammation are known to increase the risk of cancer [1,3]. Obesity is one such condition, and is a major avoidable risk factor in the development and progression of multiple cancers. The accumulation of adipose tissue as a result of hypernutrition and/or nutritional imbalance is associated with a chronic low-grade systemic inflammatory state [4].

Persistent inflammation is at the core of chronic liver disease (CLD), which can arise from multiple etiologies. Hepatitis B/C chronic viral infection accounts for the majority of cases globally, however diet and alcohol-related liver damage are intensifying as major risk factors in the development of CLD, particularly in countries with high socio-economic status [5]. The Western lifestyle (high calorie/low fiber diet and physical inactivity) which is becoming ever more prevalent across the globe, is associated with obesity and the manifestation of a systemic inflammatory state, which presents in the liver as non-alcoholic fatty liver disease (NAFLD) [6].

The prevalence of NAFLD has risen markedly in recent decades with epidemiological data estimates pointing to a staggering 25.24% of the global population affected [7]. NAFLD encompasses a spectrum of clinical conditions ranging from hepatic steatosis to severe inflammatory steatohepatitis (NASH) [7], and it is estimated that 25% of patients with NAFLD progress to NASH [8]. Alcoholic liver disease (ALD) also represents a spectrum of disease severities ranging from mild steatosis to severe hepatitis. Although distinct, the pathogenesis of both NAFLD and ALD is largely driven by activation of inflammatory pathways, with innate and adaptive immune cells, as well as non-immune cells within the liver engaging cooperatively in inflammatory responses during CLD [9]. In progressive NAFLD and ALD, inflammatory activation of liver-resident fibroblasts known as hepatic stellate cells (HSCs), promotes fibrosis which ultimately precedes the onset of cirrhosis (end-stage liver disease) [10]. Cirrhosis itself was recently reported as the 12th leading cause of death in the United States (US) and importantly, cirrhosis is the strongest risk factor for the development of hepatocellular carcinoma (HCC), the most common form of primary liver cancer [11,12,13].

HCC represents approximately 80% of primary liver cancer cases, and is currently the 6th most frequently diagnosed cancer worldwide [12]. More so than other cancers, chronic inflammation is a hallmark of HCC, with 90% of diagnoses occurring in the context of CLD [14]. Intrahepatic cholangiocarcinoma (iCCA) is a malignancy of bile duct cells and is the second most common form of primary liver cancer. Inflammatory pathologies such as liver fluke infection and primary sclerosing cholangitis are also major risk factors for the development of this aggressive disease which affects approximately 1 per 100,000 of the US population [15].

Inflammation within the liver also plays a huge role in the generation of a ‘pro-metastatic’ niche. The liver is the most common host for metastasis of other solid malignancies such as breast, lung, melanoma, and many cancers of the gastrointestinal tract including pancreatic and colorectal cancer (CRC) [16,17]. Alarmingly, twenty percent of CRC patients present with liver metastasis at diagnosis (synchronous metastasis), while two-thirds of patients will go on to develop liver metastasis during the course of their disease [18]. The five-year overall survival rate for localized tumors is 90%, however the presence of distal metastasis lowers this to a dismal 14% [19].

Given the liver is one of the most afflicted organs in cancer and the essential role of inflammation in driving liver tumors, in this review, we will address the influence of diet and alcohol in regulating inflammatory processes during liver tumorigenesis. Focus will be on HCC as the most common form of primary liver cancer (comprehensive reviews as to the role of inflammation in the pathogenesis of iCCA can be found elsewhere [20]). We will also concentrate on how dietary-induced inflammation shapes the liver microenvironment and influences CRC metastasis to the liver, given the large proportion of liver tumors they represent.

## 2. Liver Structure and Function in the Context of the Gut–Liver Axis

The liver is fundamental in metabolizing nutrients obtained via the diet as well as being central in the biosynthesis of plasma proteins and in the production and secretion of bile. Moreover, the liver serves as a detoxifier, recycling and removing unnecessary waste products and toxins [21,22].

The liver and gut are highly interconnected which is necessary to maintain homeostatic function in both organs. This bidirectional crosstalk is enabled by anatomical connections via the portal vein, systemic circulation and the biliary tract [23]. The gut communicates with the liver via the portal vein, and importantly, the liver is the first organ to receive this blood which is enriched in dietary metabolites, pattern-associated molecular patterns (PAMPs) and bile acids (BAs). The intestinal epithelial barrier is vital in the function of the gut–liver axis during homeostasis and disease by allowing selective movement of nutrients while restricting the passage of microbial pathogens [24]. Tightly adhered enterocytes covered in a mucus-rich layer surround the gut lumen. Commensal bacteria comprising the intestinal microbiome also exist at the mucosal–lumen interface, contributing to digestion of nutrients, the products of which support enterocyte function and barrier integrity [25]. In the context of impaired barrier function, micro-organisms and their PAMPs can efficiently cross the epithelial barrier, exposing the liver to these pathogens via the portal circulation [26].

Also vital in maintaining the gut–liver axis are liver-derived anti-microbials and BAs, which are delivered to the gut via the bile duct which enters the duodenum in convergence with the pancreatic duct at the Ampulla of Vater [26,27]. Within the intestine, BAs act as surfactants to enable efficient emulsification and subsequent absorption of dietary fats, lipids and vitamins [23]. BAs are also versatile signaling effectors acting largely via binding to nuclear farnesoid X receptors (FXRs). For example, FXR stimulation can suppress cholesterol 7α-monooxygenase activity, an enzyme involved in BA synthesis from cholesterol, therefore providing an auto-regulatory feedback loop [23]. Regulation may occur indirectly via circulating FGF-15 (FGF-19 in mouse) produced by enterocytes following activation of FXR in the intestine, or directly via FXR activation in hepatocytes [28]. Importantly, BAs also crosstalk with the intestinal microbiome. Due to their amphipathic nature, BAs exert damage to the bacterial membrane and the activation of FXR on enterocytes induces the secretion of antimicrobial peptides, limiting overgrowth of the gut microbial communities and acting to protect the barrier function of the intestinal epithelium [29]. The majority of bile acids (95%) are reabsorbed by enterocytes via the apical sodium dependent bile acid transporter (ASBT) and released into the enterohepatic circulation to be recycled in the liver, while a minority are metabolized by the gut microbiota. Primary BAs cholic acid (CA) and chenodeoxycholic acid (CDCA) are first deconjugated by bacterial bile salt hydrolases, and as such they cannot be transported via the ASBT but can move via passive diffusion [30]. Within the colon, non-conjugated primary BAs are subject to 7-dehydroxylation by gut microbiota, generating the secondary BAs deoxycholic acid (DCA) and lithocholic acid (LCA) [31,32]. This transformation process facilitates elimination of BAs in the feces, however a fraction of secondary BAs diffuses into the enterohepatic circulation. Primary BAs CDCA and CA bind with higher affinity to FXR than secondary BAs DCA and LCA, therefore bacterial metabolism of BAs impacts on activation of FXR and its actions, which also include regulation of glucose and lipid homeostasis, insulin signaling and immune activation [30].

### 2.1. Parenchymal Liver Cells

Hepatocytes perform diverse synthetic, metabolic and detoxifying roles in the liver, processing endogenous and exogenous (dietary-derived) biomolecules. Hepatocyte gene expression profiles are dependent on their proximity to portal and central circulation, ultimately resulting in metabolic zonation across the lobule [33,34]. This partitioning of function enhances the efficiency of liver output by a distribution of labor, however is dynamic in response to oxygen availability and external morphogens [34]. Hepatocytes synthesize many plasma proteins including albumin and clotting factors, as well as endocrine factors such as insulin-like growth factor 1 (IGF-1). Integral to function is the expression enzymes facilitating the metabolism of dietary-derived carbohydrates, lipids, amino acids and vitamins. Hepatocytes also supply primary BAs generated by cholesterol metabolism to the gut. Primary BAs, cholic acid and chenodeoxycholic acid are conjugated with glycine or taurine prior to secretion in the bile, where they are delivered to the intestine via the bile duct. Cholangiocytes form the bile duct and predominantly function in modifying the bile [35]. Bile is composed of BAs, bilirubin, phospholipids, cholesterol, water and electrolytes, and is a route of elimination of toxins from the liver [36]. Cholangiocytes sense bile composition using cilia and respond by secretory and absorptive processes to dilute and alkalinize bile flow [37].

### 2.2. Non-Parenchymal Liver Cells

Liver sinusoidal endothelial cells (LSECs) line the liver microvasculature (sinusoids), providing a barrier between the hepatic blood flow and the hepatocytes. LSECs characteristically possess open fenestrae and unique in comparison to other endothelial cells, they lack an underlying basement membrane. These open fenestrae are 50–150 nm wide pores, which cluster together to act as a two-way channel for the flow of soluble molecules through the endothelial barrier between the blood and hepatocytes [38]. LSECs are also highly efficient scavengers, capable of clearing macromolecules, such as stabilin bound oxidized low density lipoprotein, by endocytic receptor-uptake and trans-cytosis to the Space of Disse, further contributing to the delivery of metabolic substrates from the blood to hepatocytes [39,40].

Since the liver directly receives and processes antigen-enriched blood from the portal circulation and is therefore highly susceptible to the influx of pathogens, the liver has a central role in immunity [41]. Towards this function, LSECs provide an adhesive niche for myeloid cells and lymphocytes and the unique structure of LSECs allows naïve lymphocytes to contact hepatocytes via cytoplasmic extensions through LSEC fenestrations [41,42]. LSECs also contribute towards regulation of T-cell activation and tolerance through their antigen-presenting capabilities [39]. Further contributing to the immunological function of the liver are Kupffer cells (KCs). Part of the reticuloendothelial system, these liver-resident macrophages patrol the sinusoidal blood under physiological conditions, acting as a filter to eliminate gut-derived bacteria and microbial by-products, as well as phagocytosing spent erythrocytes [43]. Within the sinusoid, KCs interact with lymphocytes, and are key in promoting T-cell tolerance to prevent immune responses to harmless antigens, which is imperative given the continuous exposure to innocuous antigens from the gut [44]. A wide repertoire of tissue-resident lymphocytes are found in the liver including conventional CD4+ and CD8+ T-cells, B cells and natural killer cells (NK cells) and non-conventional γ δ T-cells and innate natural killer T cells (NKT cells) [41,45]. Together, these hepatic lymphocytes, along with antigen-presenting LSECS, Kupffer cells and dendritic cells, confer both innate and adaptive defense and importantly, tolerance within the antigen-rich hepatic microcirculation. HSCs, the liver-resident fibroblasts, are situated in the Space of Disse and are characterized by their storage of vitamin A. Under physiological conditions, HSCs are quiescent, however can be activated by signals from surrounding inflammatory cells to transdifferentiate to a proliferative myofibroblast capable of extensive extracellular matrix (ECM) production [46]. Although this response is critical in wound healing, incessant activation of HSCs is fundamental in the development of fibrosis, a feature of end-stage CLD [47].

### 2.3. Animal Models of HFD and Alcohol-Induced Liver Disease

Murine models of NAFLD and ALD have been invaluable in elucidating the cellular mechanisms underlying HCC development in the context of CLD, as subsequently detailed in this review. However, across rodent studies, there are inconsistencies in the use of these models, which does present a caveat in their interpretation. For example, commercial high-fat diets can range from 45% to 60% in fat content, obviously impacting on the overall macronutrient ratio [48,49]. However, the metabolic and physiological changes induced by 45% or 60% HFD are thought to be comparable [48,49,50]. The influence of the fatty acid profile of individual HFD models is also important to note. Specifically, diets high in saturated fat are more obesogenic than diets enriched in mono- and poly-unsaturated fatty acids [51]. Current models of ALD vary in ethanol dosage, route of administration and the duration of exposure, however none of these models recapitulate the full spectrum and timeline of ALD pathogenesis, including the development of fibrosis [52]. Combining ethanol feeding with a second hit such as carbon tetrachloride, a hepatotoxin, does result in liver fibrosis, but importantly, this does not reflect the pathology of human ALD [52,53]. These models require improvement in order to more accurately mimic the course of human ALD, although they have provided essential understanding as to mechanisms of alcohol-induced toxicity in the liver.

## 3. Diet, Inflammation and Liver Tumorigenesis

### 3.1. Hepatocellular Carcinoma (HCC)

The interplay between inflammation and tumorigenesis is well established, and HCC is a paradigm for this interaction given that CLD is a significant driver of HCC in virtually all cases [9,54]. In parallel with the rising NAFLD incidence, a rise in the number of HCC diagnoses is expected and by 2030, it is predicted that HCC will be the 3rd leading cause of cancer death in the United States [55,56]. In line with this, epidemiological studies have linked early-adult obesity as a significant contributor to HCC risk [57]. The role of alcohol in HCC risk is also well recognized, and high levels of alcohol-induced inflammation significantly correlate with disease severity and poor prognosis in HCC patients [58,59]. The progression from CLD to HCC is highly complex. Etiology-dependent features and mechanisms are involved; however, inflammation is central to all.

HFD and excessive alcohol consumption both initially lead to the development of hepatic steatosis, characterized by the build-up of lipid storage droplets within hepatocytes [60]. Prolonged accumulation of excess free fatty acids (FFAs) is termed lipotoxicity, resulting in alterations of membrane composition, mitochondrial dysfunction and the generation of reactive oxygen species (ROS), as well as activation of endoplasmic reticulum (ER) stress and the unfolded protein response (UPR) [61]. Together, these pathways contribute to DNA damage and hepatocyte cell death, which initiates infiltration of innate and adaptive immune cells [62]. The resultant chronic inflammation can further induce hepatocyte death, as well as compensatory proliferation and stimulate HSC activation triggering fibrosis. Ultimately, these processes lead to HCC development in the absence of effective anti-tumor immune surveillance [63].

#### 3.1.1. Stress-Related Inflammation in HCC–Oxidative Stress, ER Stress and Autophagy

In NAFLD, hepatic steatosis is primarily mediated by excess free fatty acids (FFAs) which can be released by lipolysis of adipose tissue, generated by hepatocytes in de novo lipogenesis (DNL), or obtained from the diet [64]. Insulin resistance, which is a consequence of obesity, contributes to increased levels of circulating FFAs by suppressing lipolysis, and this source represents 59% of all FFAs found in the liver of NAFLD patients [64,65]. Excess fructose intake, which is common in Western diets, strongly upregulates DNL and accounts for approximately 26% of FFAs in the liver [64,66,67]. In a choline deficient-high fat diet (CD-HFD) mouse model of NASH, activation of CD8^+^ T cells and NKT cells induced FFA uptake by hepatocytes via the lymphotoxin-β receptor and accelerated NASH to HCC transition by activating nuclear factor kappa-light-chain-enhancer of activated B cells (NF-κB) signaling in hepatocytes [68].

**Figure 1:**
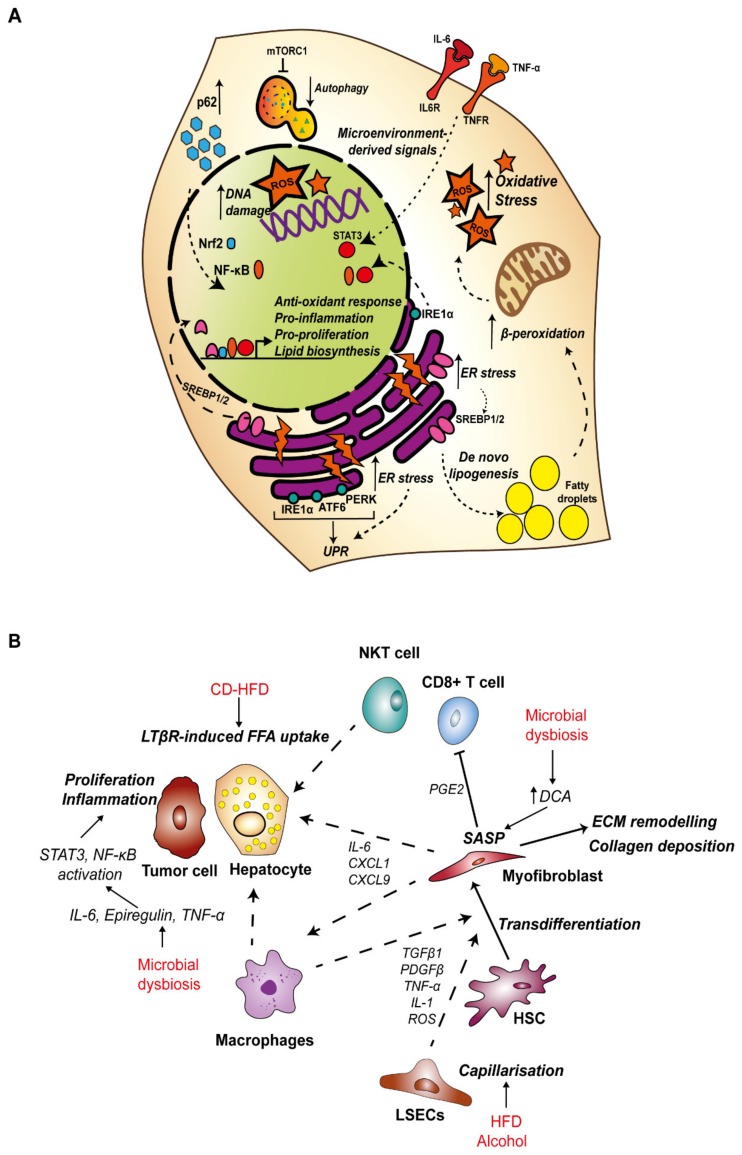
Dietary-induced inflammation during hepatocellular carcinoma (HCC) development. (**A**). Hepatocytes undergo major metabolic changes during CLD progression to HCC. This figure outlines major pathways and processes induced by HFD and alcohol in hepatocytes which contribute to the development of HCC. Generally, HFD and alcohol induce steatosis, which can be further promoted by ER stress-induced de novo lipogenesis by activation of SREBPs. ER stress engages the UPR response, and UPR effector IRE1α is required to facilitate activation of NF-κB and STAT3 signaling during NASH. Fatty acid accumulation within the cell promotes mitochondrial dysfunction and oxidative stress associated with increased ROS, which can induce DNA damage and cell death. Cytokine secretion from neighboring cells can trigger pro-inflammatory signaling and compensatory proliferation of hepatocytes. Accumulation of the autophagy substrate adaptor p62 can stabilize Nrf2 and the antioxidant response, induce pro-inflammatory NF-κB signaling and induce mTORC1 to impair the autophagy response. (**B**) Schematic illustrating cell–cell crosstalk within the liver parenchyma during HCC tumorigenesis. HFD and alcohol and associated microbial dysbiosis promote activation of pro-proliferative, pro-inflammatory, pro-fibrogenic and pro-angiogenic signaling within the liver microenvironment, and suppress the anti-tumor immune response. ATF6—activating transcription factor 6; CD-HFD—choline-deficient high fat diet; CXCL—chemokine ligand; DCA—deoxycholic acid; ECM—extracellular matrix; ER—endoplasmic reticulum; FFA—free fatty acids; HCC – hepatocellular carcinoma; HSC—hepatic stellate cell; IL—interleukin; IRE1α—inositol-requiring enzyme 1α; LSEC—liver sinusoidal endothelial cell; LTβR—lymphotoxin β receptor; NF-κB—nuclear factor kappa-light-chain-enhancer of activated B cells; NKT—natural killer T; Nrf2—Nuclear factor erythroid 2-related factor 2; PDGF—platelet derived growth factor; PERK—protein kinase R -like endoplasmic reticulum kinase; PGE2—prostaglandin E2; ROS—reactive oxygen species; SASP—senescence-associated secretory phenotype; STAT3—signal transducer and activator of transcription 3; TNFR—tumor necrosis factor receptor; UPR—unfolded protein response.

The lipid overload associated with hepatic steatosis overwhelms the metabolic capacity of hepatocytes (Figure 1A). Mitochondria adapt to excess FFAs by increasing β-oxidation, which in turn generates an excess reactive oxygen species (ROS), leading to oxidative stress and mitochondrial dysfunction [69,70]. Lipotoxicity also induces ER stress and the UPR. The ER is fundamental in the synthesis, transport and folding of proteins and the storage of calcium [71]. The ER is also the primary site for regulation of lipid homeostasis. Sterol regulatory binding protein (SREBPs), transcription factors which control expression of enzymes required for synthesis of FAs, phospholipids and cholesterol are anchored in the ER as inactive precursors in association with the sterol-binding SCAP-INSIG complex (SREBP cleavage-activating protein and insulin-induced gene). Upon cholesterol depletion, SREBP-SCAP moves to the Golgi, where SREBP1/2 are cleaved by site 1 and site 2 proteases. This facilitates translocation of the N-terminal fragment of SREBP1/2 to the nucleus for transcription of target genes [72,73]. The accumulation of unfolded or misfolded proteins and calcium imbalance induces ER stress, and importantly, ER stress can promote lipogenesis. ER stress-induced caspase-2 activates SREBP1/2 via proteolytic activation of site 1 protease to initiate fatty acid and cholesterol synthesis and drives progression to NASH in a model of HFD [74]. In a separate mouse model, a high saturated fat diet was shown to trigger hepatic ER stress and steatosis, and this was associated with increased caspase 3 activity [75,76].

Importantly, ER stress initiates the UPR pathway as a resolution mechanism. The key sensors of ER stress and mediators of the UPR response are activating transcription factor 6 (ATF6), protein kinase-like eukaryotic initiation factor 2α kinase (PERK) and inositol-requiring enzyme 1α (IRE1α), which are all located on the ER membrane, bound by immunoglobulin heavy chain binding protein (BiP). Misfolded and unfolded proteins bind to and sequester BiP, releasing the UPR sensor proteins [77]. Activation of the UPR pathways aims to rebalance equilibrium by increasing the folding capacity with extra chaperones, degrading misfolded proteins and attenuating translation [77]. However, activation of the UPR is also a source of inflammation whereby ATF6α and IRE1α are both activators of NF-κB signaling [78,79]. In a mouse model of HFD, ER stress promoted TNF-α-driven inflammatory signaling in hepatocytes and was required for HFD-induced NASH to HCC transition [80]. Furthermore, IRE1α was required for tumor necrosis factor-α (TNF-α) and interleukin-6 (IL-6)-mediated activation of signal transducer and activator of transcription 3 (STAT3), which promoted hepatocyte proliferation to drive HCC development [81]. Altogether, ER stress is necessary for the progression of NASH to HCC and this is in part due to the triggered inflammation.

Another key component of the cellular stress response is autophagy. Autophagy is activated during periods of cell stress, with the purpose of regaining balance and homeostasis by directing degradation and recycling of damaged organelles such as mitochondria, misfolded proteins, ROS and DNA damage to provide the cell with biomolecules required to maintain vital functions [82]. This process is mainly mediated by autophagy-related genes, which are required for the formation of autophagosomes and their interaction with lysosomes [83]. During the early stages of HCC tumorigenesis, the process of autophagy may be predominantly tumor suppressive, reacting to oxidative and ER stress by the removal of damaged organelles and genotoxic free radicals. Yet in advanced tumors, autophagy may facilitate cell survival during hypoxia and nutrient starvation [84]. Hypernutrition and alcohol have both been implicated in failure of the autophagy response, which may aid disease progression by the accumulation of lipotoxicity and oxidative stress [85].

p62 is an autophagy adaptor protein, which is accumulated in the early stages of HCC tumorigenesis [86]. The role of p62 in autophagy is to deliver cargo to the autophagosome for degradation, resulting in degradation of p62 in the process. Dysfunction of autophagy therefore leads to a build-up of p62 and its associated cargo [87]. Nuclear-factor-erythroid 2 p45-related factor 2 (Nrf2) is a central orchestrator of the cells’ response to oxidative stress, acting as a transcription factor to maintain redox status by binding the antioxidant response element to induce a battery of target genes, including p62 [88,89]. Activity of Nrf2 is tightly controlled by its negative regulator Kelch-like ECH-associated protein 1 (KEAP1), an E3 ubiquitin ligase substrate adaptor which targets Nrf2 for degradation [88]. Importantly, p62 binds KEAP1 at its Nrf2-binding domain and the sequestration of Keap1 by excess p62 stabilizes Nrf2 levels. By inducing p62 at the transcriptional level, Nrf2 also establishes a positive feedback loop for its own activity [89]. Nrf2 has shown to be protective against the progression of NAFLD to NASH by ameliorating oxidative stress [90,91,92,93,94], although in the context of autophagy deficient mice, stabilization of Nrf2 by p62 sequestration led to increased liver injury [95]. Mutations in *NFE2L2* (Nrf2) and KEAP1 that prevent Nrf2 degradation are two of the most frequently observed mutations in human HCC (3% and 5% of cases respectively), suggesting this pathway may be a driver of tumorigenesis in the liver [96]. NF-κB signaling is also activated by p62, and in turn NF-κB is another major regulator of p62 transcription [97,98]. p62 is also involved in activation of mammalian target of rapamycin 1 (mTORC1), a nutrient sensor which regulates cell growth and proliferation and inhibits autophagy [99,100]. Accumulation of p62 has been demonstrated in several mouse models of HCC and in human patient cohorts [80,84,86]. Deletion of p62 in combination with ER stress and HFD was sufficient to abrogate NASH-associated fibrosis and reduce tumor burden in a mouse model of HCC [86]. Mechanistically, it is thought that oxidative stress-induced Nrf2, inflammation-induced NF-κB and mTORC1 inhibition of autophagy contribute to increased levels of p62 [86,101]. This initiates a self-amplifying autoregulatory loop to further drive activation of pro-inflammatory and pro-proliferative pathways to enhance tumorigenesis. Altogether, it is apparent that ER stress is necessary for the progression of NASH to HCC, supported by presence of oxidative stress and inflammation.

The main cause of inflammation in ALD are the products and by-products of ethanol metabolism [102]. The toxicity of alcohol, the development of ALD and its association with HCC is primarily mediated by the oxidative metabolites of ethanol, in particular acetaldehyde. Two mechanisms convert ethanol to acetaldehyde in hepatocytes. Firstly alcohol dehydrogenase, and secondly the microsomal ethanol-oxidizing system (MEOS) involving cytochrome p450 2E1 (CYP2E1) which is induced following acute and chronic alcohol intake [103]. It is well established that alcohol induces hepatic steatosis. Ethanol ingestion is known to induce ER-bound SREBP1 to enhance lipogenesis [104] and downregulation of peroxisome proliferator-activated receptor-α (PPARα) signaling impairs fatty acid β-oxidation [105,106], both of which can account for the development of fatty liver.

In spite of epidemiological studies linking alcoholism to HCC, the direct and unique mechanisms underlying alcohol-induced HCC are not fully understood [107]. Molecules of acetaldehyde can react with DNA to from irreparable DNA-adducts as well as inhibit repair of DNA-damage from alkylation, therefore increasing the risk of mutagenesis which could lead to activation or deletion of oncogenes or tumor repressors respectively [108,109]. Furthermore, MEOS/CYP2E1-directed metabolism of ethanol generates vast amounts of ROS, inducing formation of lipid peroxides, which can also form adducts with DNA as well as vast cell injury and activation of cell death pathways [110]. Hepatocyte cell death can trigger compensatory proliferation of surviving hepatocytes, which can have important consequences in the expansion of mutated clones with greater fitness [107]. The pro-inflammatory cytokine TNF-α is central to the pathogenesis of ALD [111], and mouse models of alcohol feeding indicate a mechanistic role for TNF-α in the development of HCC, showing that TNFR1 activated Ras-MAPK signaling can promote ethanol-induced hepatocyte proliferation [112]. The tumor-promoting and inflammatory effects of alcohol are also concerned with its impact on the gut–liver axis, which will be discussed below.

#### 3.1.2. Dysregulation of the Gut–Liver Axis in the Development of HCC

**Figure 2:**
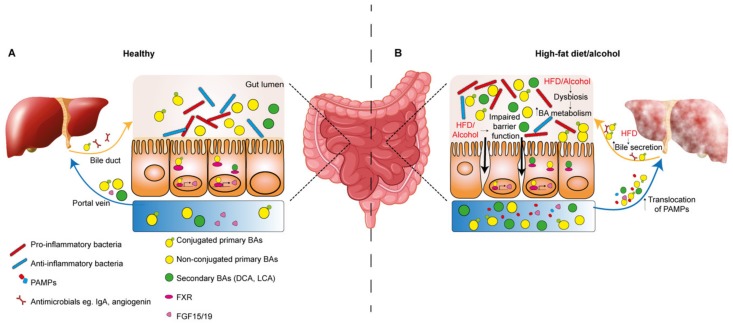
The effect of high-fat diet and alcohol on the gut–liver axis. (**A**) During normal homeostasis, liver-derived conjugated primary BAs and antimicrobials such as IgA and angiogenin are delivered to the gut via the bile duct. Conversely, via the portal vein, the liver receives gut-derived bacterial metabolites, PAMPs and recycled and metabolized BAs. BAs, which are required for emulsification and absorption of dietary fats and vitamins, are deconjugated and dehydroxylated to form secondary BAs. Primary (conjugated and non-conjugated) and secondary BAs can bind nuclear Farnesoid X receptors. For example, stimulation of FXR can induce FGF15 (or FGF19 in mouse), which negatively regulates BA synthesis in the liver. (**B**) High-fat diet (HFD) and alcohol induce dysbiosis of the gut microbiota, associated with an increase in pro-inflammatory microbial species. Up-regulation of bile secretion in response to HFD results in an increased pool of BAs in the gut lumen, which in turn are metabolized by the gut microbiota. The exact consequences of the increased metabolism of BAs by the microbiome is unknown, however secondary BAs such as DCA have pro-tumorigenic activity in the context of liver cancer. Impaired intestinal barrier function is observed in the context of HFD and alcohol, which can result in increased exposure of the liver to PAMPs in portal circulation, stimulating pro-inflammatory signaling. BA—bile acid; DCA—deoxycholic acid; FGF15/19—fibroblast growth factor 15/19; FXR—farnesoid X receptor; HFD—high-fat diet; IgA—immunoglobulin A; LCA—lithocholic acid; PAMP—pathogen-associated molecular pattern.

The intestinal microbiome is also a key mediator of hepatic inflammation in NAFLD and ALD pathogenesis as well as in HCC [113,114,115]. Importantly, diet and alcohol are linked to composition and homeostasis of the gut microbiota, and dysfunction of homeostasis can impact the gut–liver axis (Figure 2B) [115,116,117,118,119,120]. Alterations to the composition of the microbiome is termed dysbiosis, which can involve a reduction in diversity of the microbiota population, loss of particular supporting species, increased abundance of pro-inflammatory pathogenic species and a change in the metabolic capacity of the overall microbiota population [118,121].

In the context of CLD, increased hepatic exposure to bacteria and their metabolites can be attributed to impaired epithelial barrier integrity, which permits the translocation of PAMPs such as lipopolysaccharide (LPS), flagellin and leipotechoic acid (LTA) to the liver via the enterohepatic circulation [26]. Activation of pattern recognition receptors (PRRs), particularly Toll-like receptors (TLRs) on liver resident cells, induces inflammatory cytokine signaling. For example, LPS binding to TLR4 triggers activation of KCs promoting the release of TNF-α, IL-6 and transforming growth factor-β (TGF-β) [122].

Compromised barrier function has been associated with alcohol intake, such that acetaldehyde can disrupt tight junction integrity between enterocytes [123,124]. Importantly, microbiota-mediated activation of TLR4 in the chronically injured liver induced proliferation via induction of the mitogen Epiregulin, and stimulated transcription of NF-κB-regulated anti-apoptotic genes to promote HCC progression [125].

Altered BA metabolism as a result of alcohol or diet-induced dysbiosis is also key in the development of pro-tumorigenic inflammation. In a human study, a high-fat, low-carbohydrate diet was associated with changes in the gut microbiota profile towards species with high bile salt hydrolase activity and an increase in secondary BAs in the feces [126]. Furthermore, cirrhotic patients with continued drinking habits were found to have increased levels of secondary BAs which was associated with a parallel rise in colonic expression of inflammatory cytokines TNF-α, IL-1β, IL-6 and cyclooxygenase-2 (COX-2) [127]. DCA has been shown to increase permeability of the epithelial barrier, which may account for the inflammatory response [128].

Increased exposure to DCA in 2,4-dimethoxybenzaldehyde (DMBA) carcinogen treated mice maintained on a HFD induced a pro-inflammatory senescence-associated secretory phenotype (SASP) in HSCs [129]. This led to increased secretion of inflammatory cytokines IL-6, CXCL1 and CXCL9 downstream of inflammasome and IL-1β activation, and ultimately the development of HCC, which could be alleviated by antibiotic treatment or inhibition of DCA production [129]. Similarly, DCA in cooperation with LTA interaction with TLR-2 induced prostaglandin-E2 (PGE2) secretion from HSCs which suppressed the CD8+ T-cell anti-tumor immune response [130]. Secondary BAs have also been linked to HCC pathogenesis in the context of a NASH-inducing high-cholesterol, high-fat diet, whereby they induced mTOR signaling in hepatocytes. This could be alleviated by antibiotics, demonstrating the significance of the microbiota processed BAs in this response [131]. Bacterial fermentation of undigested dietary nutrients in the gut is also important to consider in the context of HCC [132]. Mice fed on an HFD supplemented with soluble fibers unexpectedly induced microbiota dysbiosis as well as the development of cholestatic injury induced HCC. Inhibiting the pathways involved in the fiber fermentation process or ablation of the gut microbiome with antibiotic treatment protected from HCC development [133]. Blocking re-circulation of BAs to the liver with cholestyramine also prevented HCC development, suggesting a pathogenic role for BAs in this context [133].

In recent study, bacterial metabolism of dietary fructose, obtained from fruits and increasingly from processed foods enriched in high-fructose corn syrup, was shown to enhance DNL. In a dual mechanism, dietary fructose promoted transcriptional up-regulation of DNL genes, while bacterial metabolism of excess fructose into the short-chain fatty acid acetate in the intestine, provided a source of acetyl-CoA in hepatocytes to feed DNL [67].

#### 3.1.3. Inflammation and Fibrosis in HCC

Eighty to ninety percent of HCC diagnoses are made in patients with end-stage liver disease with the presence of fibrosis, regardless of disease etiology [134]. Excessive deposition of collagen and other ECM proteins ultimately impedes proper liver function, culminating in the final pathological state of cirrhosis [135,136]. The fibrotic microenvironment does however facilitate HCC tumorigenesis whereby increased tissue stiffness has been shown to support cell survival and proliferation which can promote tumorigenesis (Figure 1B) [135]. The ECM also serves as a signaling source, for example, activation of integrin-αv can induce TGF-β signaling which in turn drives the fibrogenic function of HSCs [137].

Inflammatory cytokines, including TGF-β, platelet-derived growth factor-β (PDGF-β), TNF-α and IL-1 as well as ROS generated within the microenvironment in the early stages of liver disease activate HSCs to proliferate and differentiate into fibrogenic myofibroblasts (Figure 1B). As discussed, many factors such as gut-derived PAMPs can induce inflammation and KC activation in response to HFD and alcohol. Given KC-derived cytokines are one of the key sources of HSC activation, poor diet and alcohol can indirectly affect HSCs, driving fibrosis and ultimately the risk of HCC development. HSCs derived from the livers of rats fed a HFD and ethanol showed greater proliferation rates and levels of collagen deposition in vitro, mediated by KC-derived TGF-β1 [138,139].

LSECs characteristically possess open fenestrae and lack a basement membrane, key features which permit dynamic exchange across the sinusoidal barrier. Differentiation, or capillarization, of the sinusoidal endothelium was necessary for NAFLD-NASH progression in mice fed a choline-deficient, L-amino acid-defined or a HFD [140]. Moreover, capillarization is necessary for the development of cirrhosis, such that the sinusoidal endothelium in cirrhotic rat livers is defenestrated and an underlying basal lamina is present [141,142,143]. Defenestration of LSECs has been reported in response to alcohol feeding in mice, however in the same study, HFD did not induce changes in fenestration [144]. During NAFLD, LSECs also displayed a pro-inflammatory phenotype, which is central to the activation of HSCs and KCs in the development of fibrosis [140,145].

### 3.2. Colorectal Cancer Liver Metastasis

The gut–liver axis is critical for normal function and physiology of the digestive system, but their anatomical connection can also facilitate metastasis. Infiltration of CRC tumor cells via the portal vein can account for the trafficking and retention of metastatic CRC cells in the liver [18,146], yet their capacity to establish secondary tumors is dependent on interaction with particular liver-specific microenvironmental factors which can both hinder and facilitate metastatic seeding and outgrowth [147]. As in the initiation and progression of HCC, inflammation, particularly in the context of HFD and alcohol, has important consequences in the development of metastasis in the liver. Environmental and modifiable risk factors contribute to greater than half of CRC cases (>55%) [19]. In particular, obesity is a major risk factor for the development and progression of CRC, with obese individuals presenting with an increased risk of CRC-specific mortality [148]. Furthermore, the presence of fatty liver is associated with hepatic recurrence in CRC patients [149,150].

CRC metastasis begins with primary tumor cell detachment and intravasation into the circulation. Importantly, there is an enrichment of circulating tumor cells in the mesenteric circulation rather than the systemic circulation, meaning the liver is the first filter for these cells [151,152]. Upon reaching the tortuous liver microvasculature, large tumor cells physically arrest within the liver sinusoid where they primarily encounter LSECs, KCs and lymphocytes, inducing a pro-inflammatory response from these cells [153]. This is a key rate-limiting step in the development of metastasis, as few cells are able to survive this stress. Despite the inefficiency, a minority of surviving cells are able to extravasate through the specialized endothelial barrier to enter the liver parenchyma. Here, tumor cells can enter a dormant state, or undergo proliferation to form an avascular micrometastasis [154]. Recruitment and activation of stromal cells further contributes to the pro-metastatic niche, and drives angiogenesis to provide the metastasis with an adequate blood supply. This progression from micro- to macrometastases is further fueled by pro-tumorigenic growth factors secreted by neighboring hepatocytes, HSCs and tumor-associated macrophages (TAMs) [155,156]. Finally, liver metastatic tumors infiltrate a large proportion of liver parenchyma, compromising the normal function of the organ and ultimately increasing the risk of mortality [18]. To understand the role of dietary-induced inflammation and its influence on liver-resident cell types within the metastatic niche, we will divide the metastatic process into two differential phases; the early microvascular phase, and the extrasinusoidal phase, encompassing growth and propagation within the liver parenchyma.

#### 3.2.1. Inflammation in the Microvascular Phase of CRC Liver Metastasis

**Figure 3:**
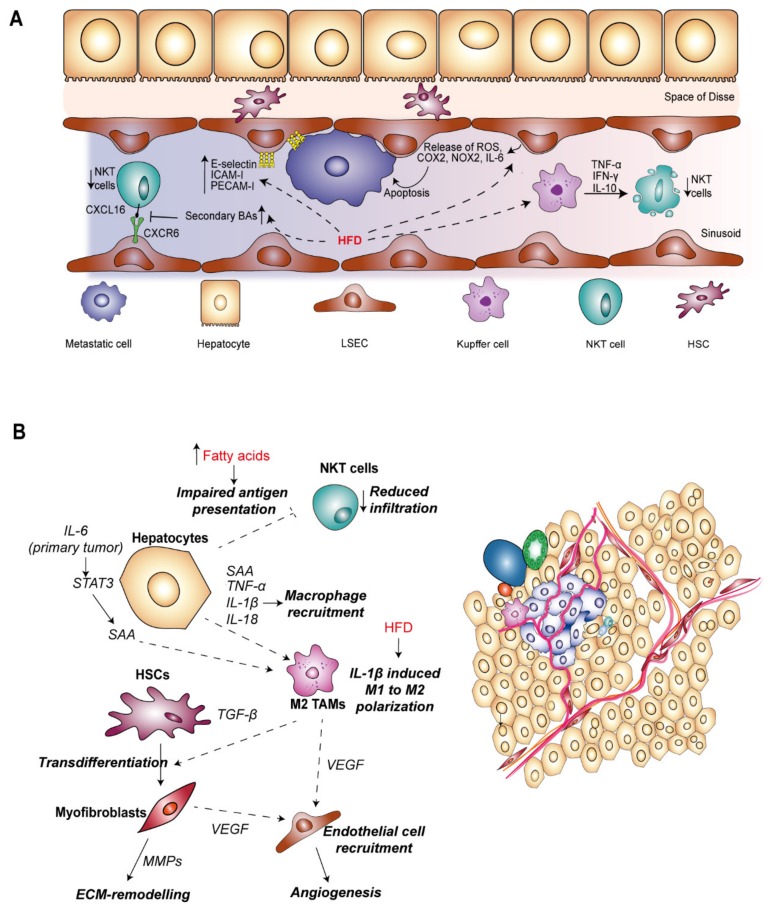
Dietary-induced inflammation during metastatic tumorigenesis in the liver. (**A**) Inflammation during the microvascular phase of CRC liver metastasis. CRC tumor cells which arrest within the liver sinusoid must evade immune cell killing and attach to LSECs in order to invade the liver parenchyma. HFD-induced inflammatory and cytotoxic mediators can promote tumor cell death within the liver sinusoid. On the other hand, HFD-feeding can impair the anti-tumor immune response by reducing NKT cell infiltration. Increased secondary BAs inhibit NKT–LSEC interaction and NKT cell adhesion by reducing the levels of CXCL16 expression. Additionally, activation of KCs, for example by translocated LPS from the gut, can induce overactivation and apoptosis of NKT cells. HFD can also promote expression of cell adhesion molecules including E-selectin and ICAM-1 by LSECs which are necessary for CRC tumor cell adhesion and extravasation. (**B**) Inflammation during the extravascular phase of metastatic tumorigenesis in the liver. Within the liver parenchyma, metastatic cells primarily encounter hepatocytes, TAMs and hepatic stellate cells. The schematic illustrates the effect of HFD on liver resident cells and the crosstalk between them which supports metastatic tumor growth. BAs—bile acids; COX2—cyclooxygenase 2; CXCL16—chemokine ligand 16; CXCR6—chemokine receptor 6; ECM—extracellular matrix; ICAM-1—intercellular adhesion molecule 1; IFN-γ—interferon-γ; IL—interleukin; KC—Kupffer cell; LSEC—liver sinusoidal endothelial cell; MMP—matrix metalloproteinase; NKT—natural killer T; NOX2—NADPH oxidase 2; SAA—serum amyloid A; STAT3—signal transducer and activator of transcription 3; TAM—tumor-associated macrophage; TNF-α—tumor necrosis factor-α; TGF-β—transforming growth factor–β; VEGF—vascular endothelial growth factor.

Resident cells within the liver sinusoid have both anti- and pro-metastatic roles, the balance of which determines the fate of tumor cells during the first step of metastasis (Figure 3A). Interruption of blood flow by tumor cell obstruction of the sinusoidal vasculature can lead to transient ischemia which induces tumoricidal inflammation owing to the release of ROS, nitric oxide (NO), TNF-α and interferon-γ (IFN-γ) by LSECs and KCs [153,155,157]. Similarly, HFD in mice also increased the levels of reactive ROS produced from LSECs [158], and increasing NAFLD severity was associated with a pro-inflammatory gene expression signature in LSECs which included increased COX-2, NOX-2 and IL-6 levels [145]. HFD-induced CLD could therefore potentiate elimination of disseminated tumor cells invading the liver sinusoid.

The activation of KCs is predominantly tumoricidal towards invading tumor cells in the context of the microvascular environment whereby they release a plethora of cytokines, leading to recruitment and activation of innate immune cells. For example, IL-12 and IL-18 secreted from KCs can stimulate expansion of NKT cells which then promotes apoptosis of tumor cells by release of perforin and granzyme [159,160,161]. Supporting this role, infiltration of NKT cells is associated with greater survival outcome in patients with colorectal liver metastasis [162]. Importantly, mice fed a HFD show reduced numbers of NKT cells in the liver [163,164,165,166], suggesting that poor diet and obesity could promote liver metastasis. HFD-induced secretion of TNF-α, IFN-γ and IL-10 from KCs promoted overactivation and apoptosis of NKT cells [163]. Infiltration of NKT cells is also mediated by their adhesion to LSECs, and this interaction is key in suppressing metastasis development [167]. Gut microbiota-mediated metabolism of primary BAs to secondary BAs reduced the expression of CXCL16 on NKT cells, a ligand for CXCR6 expressed on LSECs, and antibiotic ablation of the gut microbiota inhibited the outgrowth of liver metastasis. Although the cell lines used in this study were not of CRC origin [167], it is likely that similar processes are in play during the process of CRC liver metastasis.

In contrast, the pro-inflammatory microenvironment generated in the initial response to invading can have pro-metastatic consequences. KC-derived inflammatory cytokines, in particular TNF-α and IL-1 induce expression of cellular adhesion molecules (CAMs) on LSECs including E-selectin, intercellular adhesion molecule-1 (ICAM-1), vascular cell adhesion molecule-1 (VCAM-1) and platelet endothelial cell adhesion molecule-1 (PECAM-1) [153,168,169]. Importantly, adhesion of invading cells to LSECs via E-selectin and ICAM has been well-correlated with development of CRC liver metastasis by stimulating tumor cell extravasation into the Space of Disse [170,171,172,173]. In response to a HFD and with increasing severity of NAFLD, pro-inflammatory cytokines enhanced the expression of adhesion factors E-selectin, ICAM-I and PECAM-I on LSECs [145]. Furthermore, in vitro studies suggest that omega-3 and omega-6 fats obtained from the diet may impact CRC tumor cell adhesion by modulating membrane fatty acid composition and E-selectin expression on LSECs [174], and ultimately increasing liver metastatic burden in vivo [175]. A similar mechanism was described in modulating the interaction between tumor and Kupffer cells whereby omega-3 and omega-6 fatty acid treatment decreased KC-tumor cell binding and therefore impaired phagocytosis of tumor cells [176]. Taken together, dietary-driven inflammation can impede the metastatic process within the liver sinusoid by promoting tumor cell elimination, but paradoxically enhance the potential for metastasis development by promoting crucial LSEC–tumor cell interactions.

#### 3.2.2. Inflammation in the Extravascular Phase of CRC Liver Metastasis

Following extravasation, invading tumor cells can proceed to colonize the liver parenchyma, requiring proliferation and coercion from the tumor microenvironment to initiate angiogenesis for further support of tumor growth towards the transition to macrometastases. During this phase, metastatic tumor cells encounter further liver resident cells, including hepatocytes, HSCs and TAMs (Figure 3B).

Hepatocytes, the main functional cells of the liver are an important source of pro-inflammatory signals during metastasis, cooperating with lymphocytes and myeloid cells to establish a pro-metastatic microenvironment [177]. STAT3 is a hub for multiple signaling inputs including IL-6 and IGF-I [178], controlling transcription of survival, proliferation and inflammatory related genes. IL-6 produced by primary tumor stroma induced STAT3 signaling in 80–90% hepatocytes, compared to activation in 2% hepatocytes in non-tumor bearing mice. Deletion of IL-6 led to reduced infiltration of myeloid cells in the liver as well as reduced deposition of ECM proteins during metastatic colonization [177], cementing inflammatory IL-6-STAT3 signaling as a key promoter of metastasis development in the liver.

The production of growth factors from hepatocytes, in particular IGF-1 which also activates STAT-3, has been associated with potentiation of CRC metastasis in the liver, with blockade of the pathway inhibiting liver metastasis of CRC cells lines in vivo [179]. Knockout of IGF-1 in hepatocytes in obese mice blocked the HFD-dependent increase in inflammatory mediators IL-1β, IL-18 and TNF-α and reduced macrophage infiltration, ultimately reducing the incidence of hepatic metastases [180]. Hepatocytes are also competent APCs, contributing to engagement of T-cell responses [181]. In a mouse model of NAFLD induced by feeding with saturated and monounsaturated fatty acids, endogenous antigen presentation by hepatocytes was impaired leading to a failure in activation of NKT cells [182]. Given NKT cells play an important role in anti-tumor immunity within the liver sinusoids, their absence in the more advanced phase could facilitate tumor cell survival and potentiate macrometastases development.

Although in the early phase of CRC liver metastasis, hepatic macrophages are capable of inducing tumor cell killing, these macrophage populations also elicit pro-tumorigenic functions integral to the transition to a macrometastases [161,183]. Macrophage-secreted growth factors, including TGF-β, promote tumor proliferation and remodeling of ECM, and secretion of pro-angiogenic factors and chemokines such as vascular endothelial growth factor (VEGF), inducing the recruitment and activation of fibroblasts, pericytes and endothelial cells to drive blood vessel formation within metastatic tumors [155]. Potentiation of pro-metastatic macrophage function was observed in mice fed on a high calorie diet mediated by the NOD-like receptor C4 (NLRC4) inflammasome complex [183]. Deletion of NLRC4 led to decreased IL-1β levels and a reduction in the infiltration of M2 polarized macrophages, impairing HFD-promoted outgrowth of CRC liver metastasis. As expected, IL-1 signaling blockade also reduced the liver metastatic burden in mice harboring fatty livers, suggesting it is a key effector in the metastatic niche associated with diet-induced liver damage. Furthermore, increased levels of VEGF secreted by TAMs within the fatty liver microenvironment led to increased numbers of CD31-positive endothelial cells [183]. Therefore, dietary-induced NAFLD is capable of initiating angiogenic signaling by directing polarization of macrophages toward an M2 phenotype, thereby fueling the metastatic progression. Given the role of macrophages in stimulating numerous aspects of tumorigenesis, there is interest in targeting TAMs therapeutically in HCC [184]. Strategically, blocking recruitment of or depleting TAMs has shown efficacy in eliminating HCC tumors in the liver [184,185]. Furthermore, directing macrophages from an immunosuppressive M2 phenotype towards pro-inflammatory and cytotoxic M1 polarization has also been proposed as a therapeutic strategy, with a number of compounds inhibiting HCC tumor growth in vivo [184,185].

Under physiological conditions, HSCs remain quiescent, however during times of injury become activated and are a key figure in damage resolution through the secretion of various pro-proliferative factors and remodeling of the ECM. In the absence of a resolution signal and during periods of chronic inflammation, persistent activation of HSCs contributes to the development of fibrosis in multiple liver pathologies. Unsurprisingly, HSCs are also present within CRC liver metastatic nodules, with the activated myofibroblast phenotype also associated with a metastasis-supportive niche [186,187]. Other resident cells within the hepatic microenvironment provide a source of pro-fibrogenic cytokines, in particular TGF-β, VEGF, PDGF and connective tissue growth factor (CTGF) [46], and activation of HSCs can also be directly induced by CRC cells [188]. Secretion of ECM remodeling factors from myofibroblasts such as matrix metalloproteinases (MMPs) generates a reactive stroma, permissive to tumor cell seeding and proliferation and suitable for neovascularization and angiogenesis [153,189]. Limited evidence is available for the role of diet in directly-affecting HSC function during CRC liver metastasis. However, in potentiating the pro-inflammatory function of LSECs and KCs, HFD may indirectly induce HSC activation to facilitate metastatic progression.

### 3.3. Dietary Interventions and Therapeutic Approaches in Preventing Liver Cancer Development

Given the interaction with CLD, preventative approaches aimed at maintaining a nutritionally balanced diet and moderate alcohol consumption could prove effective in reducing the burden of liver cancer. Nevertheless, owing to the rising prevalence of CLD which will give rise to future cases of HCC, and the long-latency associated with HCC development, therapeutic approaches tackling the CLD-associated inflammation is absolutely necessary. The recommended diet for NAFLD patients is a Mediterranean diet which is enriched in unsaturated fatty acids, wholegrains and antioxidants, and low in red meat and carbohydrates from sugar [190]. Mechanistically, unsaturated fatty acids such as omega-3 and omega-9 are thought to inhibit pathways involved in DNL, alleviate oxidative stress and suppress pro-inflammatory signaling. In contrast, metabolism of omega-6 polyunsaturated fatty acids has been suggested to promote inflammation via the production of arachidonic acid [190]. Nevertheless, adherence to the Mediterranean diet has been associated with reduced hepatic steatosis in short term clinical trials, and importantly, has been shown to reduce the HCC risk as well as overall cancer mortality [191].

Targeting the microbiome with antibiotics has proven beneficial in reducing tumor burden in murine models of both HCC and liver metastasis [125,129,131,133,167]. However, translating this to humans will prove problematic, given the system-wide effects of depleting the healthy gut microbiota, as well as concerns over antibiotic resistance [192,193]. Alternatively, manipulating the gut microbiota through the diet with probiotics can alleviate inflammation. Probiotics are cultures of live microbes, typically Lactobacilli or Bifidobacteria, which can be found naturally in the diet. The premise of using probiotics is to replace pro-inflammatory bacteria with anti-inflammatory bacteria, and their use has been widely implemented to maintain normal gut flora as well as in the treatment in a number of diseases [194]. Excitingly, probiotics have shown efficacy in alleviating NAFLD in rodent models as well showing promise in human studies. This was predominantly mediated by improved intestinal barrier integrity, reduced translocation of bacterial products via the gut–liver axis, and by regulating inflammation in the liver [195,196]. The positive effects of probiotics on NAFLD have been demonstrated to impair downstream development of HCC in mouse models [197], although the clinical impact on HCC patients has yet to be established [198]. Recent efforts to target the microbiome with bacteriophages have proved promising in the context of ALD [199]. Increased levels of cytolytic *Enterococcus faecalis* were observed in the feces of alcoholic hepatitis patients correlating with poor disease prognosis [199]. Treatment of humanized mice with bacteriophages targeting *Faecalis* led to precise editing of the gut microbiome and a reduction in alcohol-induced inflammation, steatosis and fibrosis [199]. Given the specificity of phages, this approach presents an exciting therapeutic opportunity towards rewiring a dysbiotic gut microbiome in the treatment of CLD.

Activation of FXR, the nuclear BA sensor has been reported to protect against liver inflammation and fibrosis [200,201]. Interim analysis of an ongoing clinical trial involving the BA analog and FXR agonist obeticholic acid, have provided the first positive phase 3 trial outcomes in the treatment of NASH, in terms of reduction in hepatocyte ballooning and inflammation and improvements in fibrosis when compared to placebo [202].

## 4. Conclusions

Inflammation is central to the pathogenesis of HCC and is also crucial in establishing a pro-metastatic niche for the seeding and propagation of metastasis in the liver. It is clear that a major driver of inflammation in this context is the consumption of a high-fat Westernized-style diet and increased alcohol intake. Although epidemiological studies in humans suggest strong links between HFD and alcohol as factors in development of CLD, confounding factors such as co-morbidities and smoking have made it difficult to provide a definitive causal link. Preclinical models have been critical in elucidating the effects of excessive fat and alcohol intake in the liver at cellular and molecular levels. However, detailed reporting and consistent implementation of human-relevant HFD models in particular will be imperative in furthering our understanding of specific dietary factors in the development of CLD and the implications for HCC pathogenesis. This is particularly relevant given that modification of lifestyle choices, such as adherence to a Mediterranean diet, is proposed as a therapeutic strategy in patients with NAFLD. Reducing the incidence of inflammatory CLD, and consequently the risk and burden of both primary and secondary liver cancer is pertinent given the current incidence and mortality rates of these malignancies.
